# Supplementary material for: DMRforPairs: identifying Differentially Methylated Regions between unique samples using array based methylation profiles
Source: BMC Bioinformatics. 2014 May 15;15:141. doi: 10.1186/1471-2105-15-141 (PMC4046028; doi:10.1186/1471-2105-15-141)
Supplement: Additional file 2 — DMRforPairs output for the comparison of A431-MCF7 and NA17018-NA17105. Please start from the HTML files in each folder. Available via the BMC Bioinformatics website. [file 1471-2105-15-141-S2.zip › 1394847754114233_MOESM2_ESM/A431_MCF7/figures/4310.pdf]

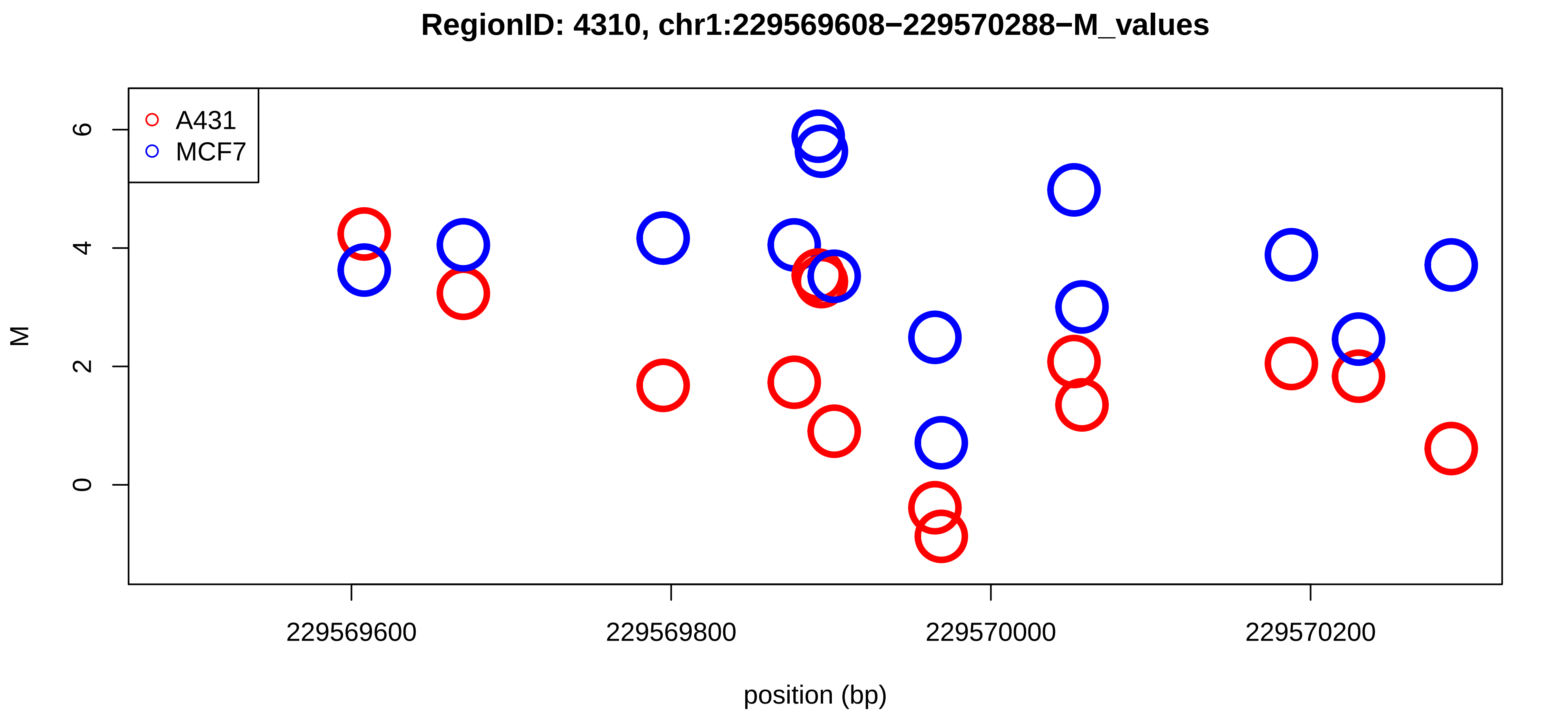

RegionID: 4310, chr1:229569608−229570288−M_values
A431
MCF7
M
0
2
4
6
229569600
229569800
229570000
229570200
position (bp)

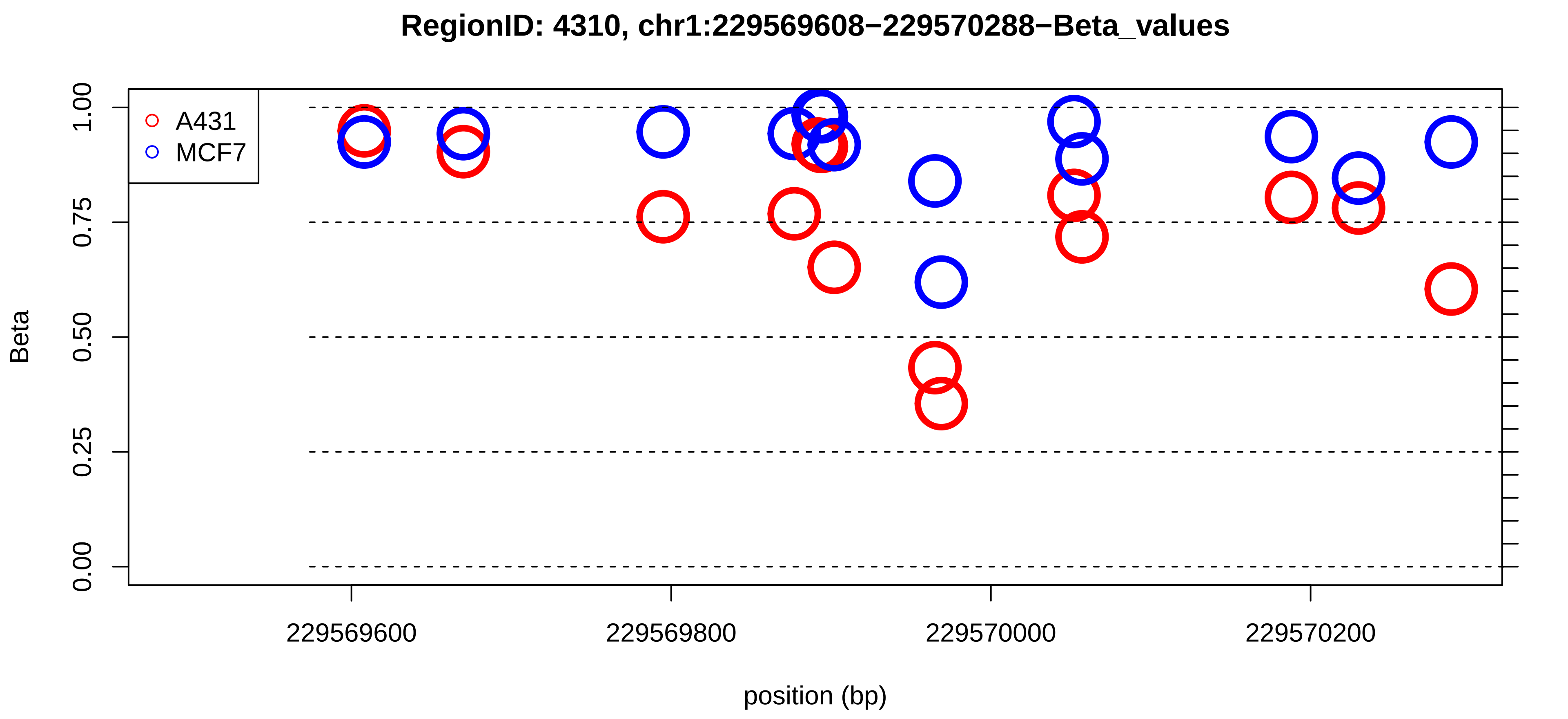

RegionID: 4310, chr1:229569608−229570288−Beta_values
A431
MCF7
Beta
0.00
0.25
0.50
0.75
1.00
229569600
229569800
229570000
229570200
position (bp)

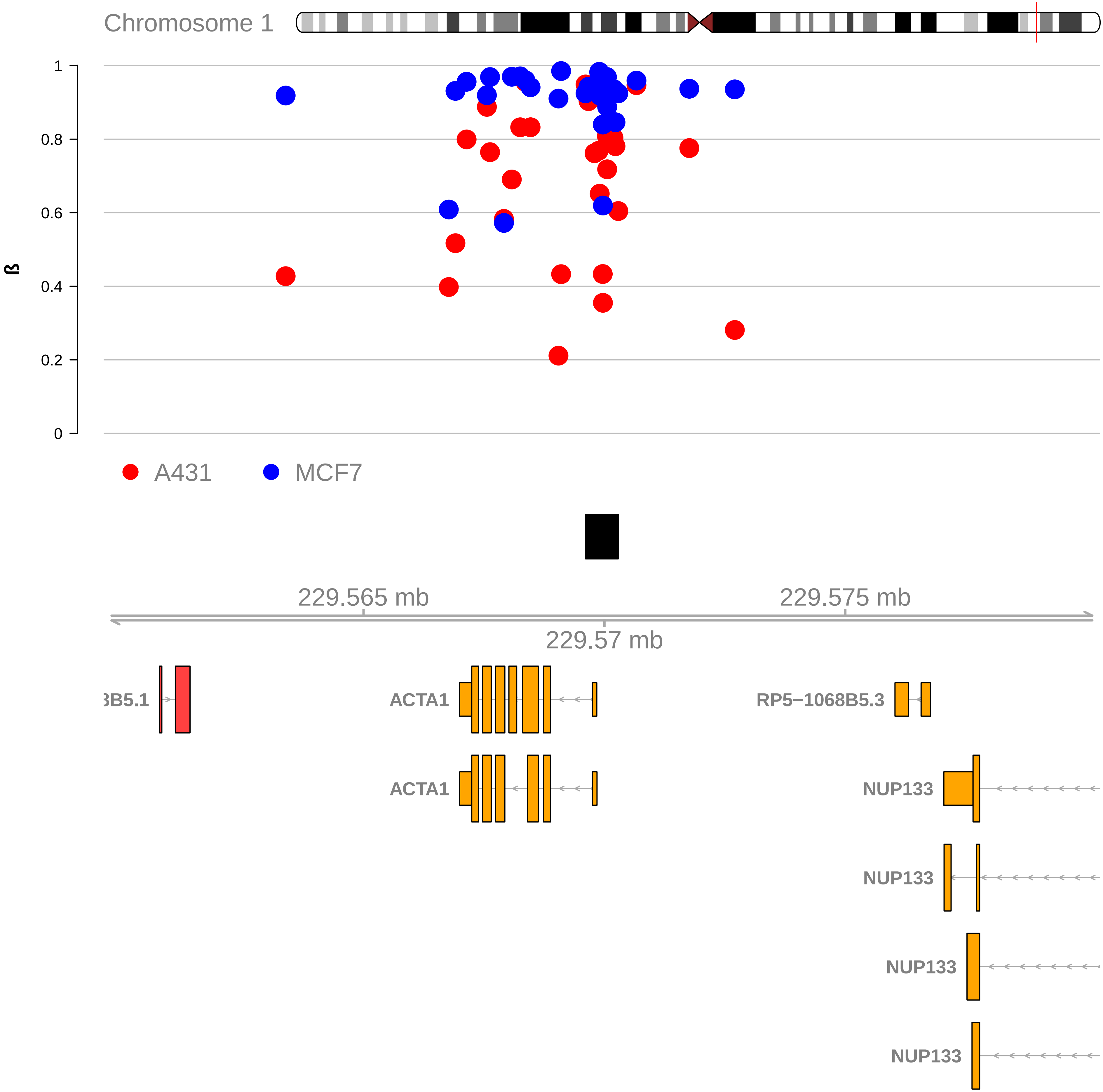
Chromosome 1
1
0.8
0.6
0.4
0.2
0
ß
A431
MCF7
229.565 mb
229.575 mb
229.57 mb
B5.1
ACTA1
RP5−1068B5.3
ACTA1
NUP133
NUP133
NUP133
NUP133
